# Improved simplicity and practicability in copper-catalyzed alkynylation of tetrahydroisoquinoline

**DOI:** 10.1007/s00706-016-1877-5

**Published:** 2016-12-09

**Authors:** Birgit Gröll, Patricia Schaaf, Michael Schnürch

**Affiliations:** Institute of Applied Synthetic Chemistry, TU Wien, Getreidemarkt 9/163, 1060 Vienna, Austria

**Keywords:** Alkynes, Catalysis, Cross dehydrogenative coupling, Oxidative coupling, Copper catalysis

## Abstract

**Abstract:**

Alkynylation reactions of N-protected tetrahydroisoquinolines have been performed using several different protocols of cross dehydrogenative coupling. Initially, a CuCl-catalyzed method was investigated, which worked well with three different N-protecting groups, namely phenyl, PMP, and benzyl and *t*-BuOOH as oxidant in acetonitrile as solvent. The peroxide could then be replaced by simple air and acetonitrile for water, leading to an overall very environmentally friendly protocol. Finally, a decarboxylative alkynylation protocol starting from alkynoic acids was also developed using again air as oxidant. This avoids the use of gaseous alkynes in the introduction of short-chained alkyne substituents.

**Graphical abstract:**

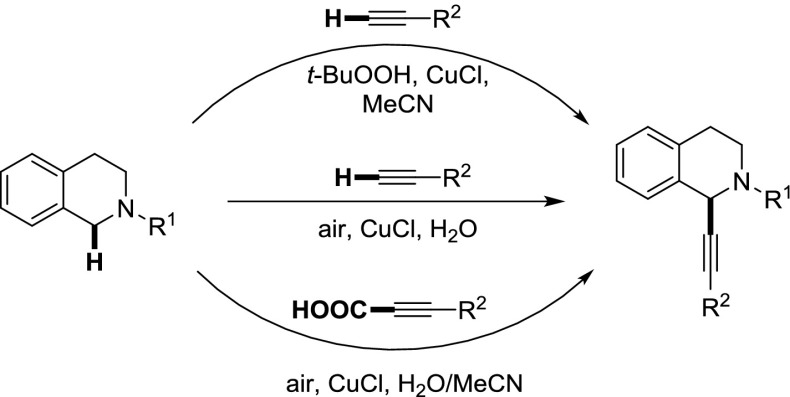

## Introduction

**Fig. 1:**
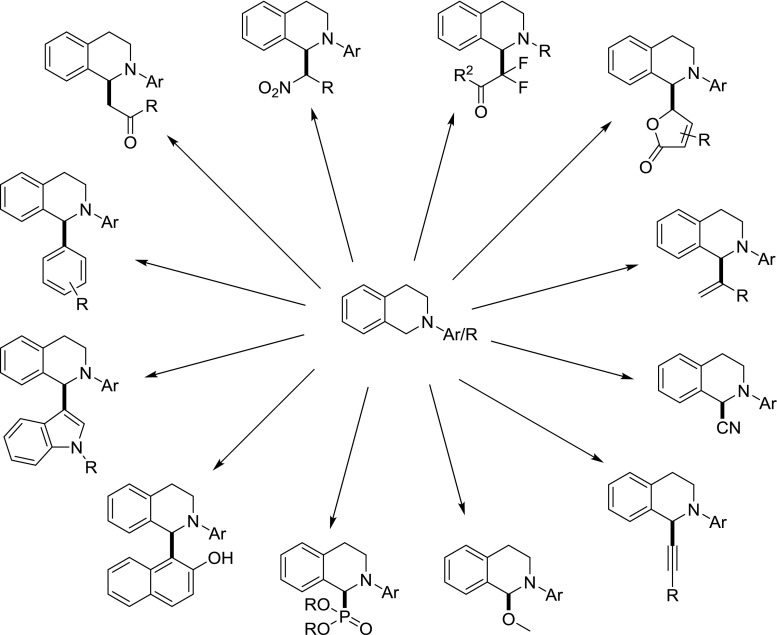
Reported CDC reactions on N-substituted TIQs

The formation of carbon–carbon bonds is key to the assembling of complex organic molecules. Hence, the development of efficient methods to make these bonds is an infinite research area. The field of metal catalysis was able to contribute significantly to this area in the last few decades. The first transformations to come into mind are arguably the famous cross-coupling reactions [1, 2]; however, in more recent times significant competition came from the field of metal-catalyzed C–H activation chemistry [3–9], where the C–H bond is exploited as functional group, replacing either the organometal or the halide part of a classical cross-coupling reaction. Even more desirable would be methods, which take advantage of a C–H bond in both coupling partners for C–C bond-forming processes, leading formally only to an equivalent of H_2_ as waste. One such method has gained prominence under the time of cross-dehydrogenative coupling (CDC) [10–12]. One substrate which plays a predominant role in CDC reactions is tetrahydroisoquinolines (TIQ). N-Substituted TIQs have been applied in a number of transformations to introduce various substituents to C1 (Fig. 1) [13–20].

The reported protocols have several common features: (1) the TIQ nitrogen carries a protecting group, mostly phenyl; (2) an external oxidant is required (mostly *t*-BuOOH); (3) the reactions are either carried out neat or in organic solvents (whereas “neat” reactions typically use a *t*-BuOOH solution in decane as oxidant).

Even though these are not severe limitations, it leaves room for improvement. A cleavable N-protecting group would definitely be an advantage since it would allow more flexible further elaboration of the CDC products. Replacing *t*-BuOOH by a more benign oxidant (ideally air) and the typically applied organic solvents by water would further improve the practicability of this approach.

As test reaction, we identified the CuOTf-catalyzed alkynylation originally published by the group of Li (Scheme 1) [21]. In this paper, even an enantioselective CDC reaction was disclosed taking advantage of chiral PyBOX-type ligands. It can be considered as a typical CDC example which uses commonly applied *t*-BuOOH as oxidant in THS as solvent.

**Figure:**
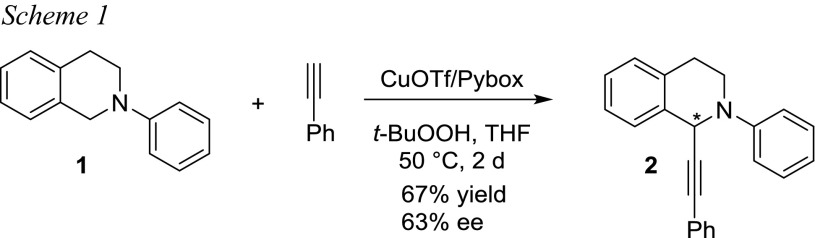

Within this contribution, we report our efforts to develop this transformation toward a more environmentally benign alkynylation method for various TIQs.

## Results and discussion

Based on the original report by the group of Li (Scheme 1) [21], we started optimizing the protocol. Li optimized his procedure toward maximum ee and in this regard he identified CuOTf as an ideal metal source giving 67% isolated yield and 63% ee for the preparation of **2** starting from *N*-phenyl-TIQ (**1**) using phenylacetylene as coupling partner. Typically, the alkyne was used as limiting reagent with 2 equivalents of **1** as coupling partner. In our case, we were not focused on enantioselective reactions, but on improving the practicability of the protocol. Hence, we started with a screening to test whether CuOTf would also be the best metal source if the main focus lies on yield rather than ee. Using again **1** as substrate and phenylacetylene as alkyne, we screened for the ideal combination of catalyst, temperature, and solvent. Reactions were carried out with CuBr, CuCl, CuCN, Cu(NO_3_)_2_·3H_2_O, (CuOTf)_2_ toluene complex, and Fe(NO_3_)_2_·9H_2_O as catalysts, at 50 and 100 °C, and in THF, acetonitrile, dichloromethane, or neat. Fe(NO_3_)_2_·9H_2_O was included as potential catalyst since we successfully applied it in indolation reactions of tetrahydroisoquinolines derivatives [19, 20]. Parameters and yields of successful experiments are listed in Table 1. It has to be mentioned that in all those experiments, a 2:1 ratio between **1** and phenylacetylene was used. This is necessary since oxidation in position 1 of TIQ is a common side reaction which cannot be suppressed completely. Hence, the alkyne is used as the limiting reagent.

**Table 1:** Parameter screening in the alkynylation of **1** with phenylacetylene

Entry	Catalyst	*T*/°C	Solvent	Yield **2**/%^a^
1	(CuOTf)_2_ toluene complex	50	THF	55
2	(CuOTf)_2_ toluene complex	50	MeCN	75
3	(CuOTf)_2_ toluene complex	50	Neat	26
4	(CuOTf)_2_ toluene complex	50	DCM	33
5	(CuOTf)_2_ toluene complex	100	Neat	n.c.
6	(CuOTf)_2_ toluene complex	100	MeCN	n.c.
7	CuBr	50	Neat	66
8	CuBr	50	DCM	53
9	CuBr	50	MeCN	55
10	CuBr	50	THF	51
11	CuBr	100	Neat	100
12	CuBr	100	MeCN	59
13	Cu(NO_3_)_2_·3H_2_O	50	Neat	80
14	Cu(NO_3_)_2_·3H_2_O	50	MeCN	61
15	Cu(NO_3_)_2_·3H_2_O	50	DCM	67
16	Cu(NO_3_)_2_·3H_2_O	50	THF	48
17	Cu(NO_3_)_2_·3H_2_O	100	Neat	72
18	Cu(NO_3_)_2_·3H_2_O	100	MeCN	37
19	CuCl	50	THF	70
20	CuCl	50	MeCN	86
21	CuCl	50	Neat	67
22	CuCl	50	DCM	65
23	CuCl	100	Neat	58
24	CuCl	100	MeCN	60
25	CuCN	50	MeCN	45
26	Fe(NO_3_)_2_·9H_2_O	50	MeCN	n.c.
27	Fe(NO_3_)_2_·9H_2_O	100	Neat	n.c.

Standard screening conditions: 0.4 mmol **1**, 0.2 mmol phenylacetylene, 0.22 mmol *t*-BuOOH (~5.5 M solution in decane), 10 mol% catalyst, and 1 cm^3^ solvent, argon atmosphere

^a^Isolated yield

It can be seen that in most cases, moderate to high yields were obtained with copper catalysts, the exception being the (CuOTf)_2_-catalyzed reactions at 100 °C, where no conversion could be detected. Also, Fe(NO_3_)_2_·9H_2_O gave no conversion at all. The solvent THF was identified as an ideal solvent to obtain high ee in the original publication of Li [21]. In our screening, the best yields were obtained either under neat conditions or using acetonitrile as solvent, depending on the copper catalyst. (CuOTf)_2_ toluene complex and CuCl gave the best yield in acetonitrile, whereas CuBr and Cu(NO_3_)_2_·3H_2_O gave better results under neat conditions. It has to be mentioned that “neat” means no addition of additional solvent, but the oxidant in the screening is provided as 5.5 M solution in decane.

The two best performing protocols were then used in a second screening in which the N-protecting group was varied. In most literature examples of cross-dehydrogenative coupling reactions, a phenyl group is attached to the TIQ nitrogen [13–18, 21]. This has to be considered as a permanent group. Cleavage of an *N*-phenyl group has been reported, but not on a TIQ substrate and only under very harsh conditions (100 equiv of Li/NH_3_/THF/40 °C, 3 h) [22]. We could show that Boc can be used instead in the CDC reaction of TIQ and indoles [19, 20]. Hence, we tested whether also in the alkynylation protocol, other N-protecting groups can be applied. The two best conditions identified in Table 1 were used on different substrates (Table 2).

**Table 2:**
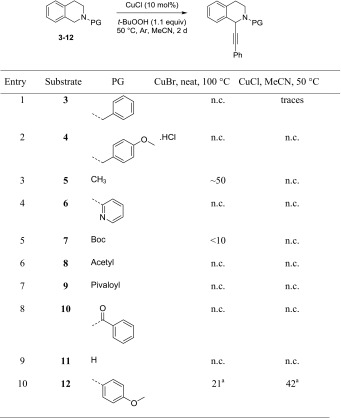
Protecting group screening

^a^GC yield with internal standard

In the case of CuBr as catalyst, at 100 °C and no additional solvent, product formation was observed for the methyl-, Boc-, and PMP-protecting groups (see Table 2, entries 3, 5 and 10, substrates **5**, **7**, and **12**). Although the yield was around 50% according to GC–MS for 2-methyl-1,2,3,4-tetrahydroisoquinoline (**5**), the alkynylation product could not be isolated due to purification difficulties even though several attempts were undertaken. Since the methyl group cannot be cleaved either, this was not further pursued. In the case of the Boc-protected substrate **7**, product formation was very low—beyond 10%—according to GC–MS and crude ^1^H NMR. In case of PMP-protected substrate **12**, the conversion remained low with 21%. In case of CuCl in acetonitrile at 50 °C, **12** gave a more promising conversion of 42% (entry 10). Trace amounts of product were detected for the benzyl-protecting group (entry 1, substrate **3**) and in all other cases no conversion was detected.

Next, the substrate scope was investigated. It was decided to use the CuCl protocol since handling of the reactions was simpler in the presence of solvent. Additionally, the PMP group gave better results in the initial screening and should be included in the substrate scope investigations.

Two alkyne coupling partners, namely phenylacetylene and 1-octyne, were the same as in the report by Li [21], so we can compare the yield between the two protocols. Otherwise, we tried to focus on alkynes which were not applied by previous reports. For phenylacetylene, we received 86% for the phenyl and 42% yield for the PMP-protecting group (Table 3, entries 1 and 8). This compares favorably to Li´s protocol in case of the phenyl PG where Li obtained 67% of **2**, but is unfavorable in case of the PMP group where Li obtained 59% yield of **14a**. In case of 1-octyne, it is just the other way round; our protocol gave higher yield for PMP (61% of **14c** vs. 48%), but lower for the phenyl PG (49% of **13b** vs 65%) (Table 3, entries 3 and 10).

**Table 3:**
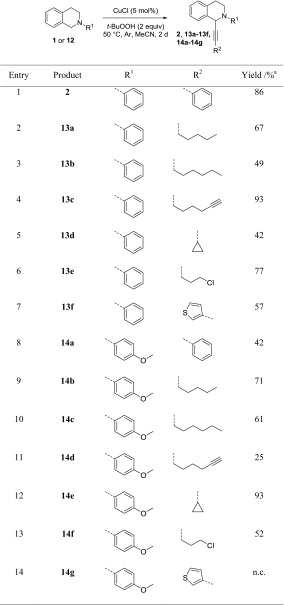
Substrate scope investigations of alkyne coupling partners

Standard conditions: 0.4 mmol **1**, 0.2 mmol alkyne, 0.22 mmol *t*-BuOOH (~5.5 M solution in decane), 10 mol% CuCl, 1 cm^3^ MeCN, argon atmosphere

^a^Isolated yield

For the other alkynes, 1-heptyne gave similar yields for both protecting groups (entries 2 and 9). For 1,7-octadiyne, an excellent yield of 93% was obtained for the phenyl PG and a low yield of 25% for the PMP group (Table 3, entries 4 and 11). Interestingly, only one of the terminal alkyne groups reacted in both cases and no products were detected in which both alkyne groups carry a TIQ residue. With 5-chloropent-1-yne, a similar but less pronounced trend was observed, with substrate **1** giving 77% yield and substrate **12,** 52%. More severe was the difference for 3-ethynylthiophene, which only gave the product in the reaction with **1** (Table 3, entry 7, 57%), but no conversion with the PMP starting material **12** (Table 3, entry 14). Finally, ethynylcyclopropane gave the highest yield in an alkynylation reaction with the PMP substrate **12** (Table 3, entry 13, 93%) and a significantly lower yield with the phenyl substrate **1** (Table 3, entry 6, 42%).

The same set of alkynes was then also reacted with the benzyl-protected substrate **3**, even though only traces of the product were detected in the reaction with phenylacetylene. It was found that the other π-system containing alkynes were equally inefficient (Table 4, entries 1, 4, and 7), but aliphatic alkynes indeed gave product formation (Table 4, entries 2, 3, 5, and 6). However, yields remained moderate to low, with the best result for ethynylcyclopropane, which gave 53% yield of **15e** (Table 4, entry 5).

**Table 4:**
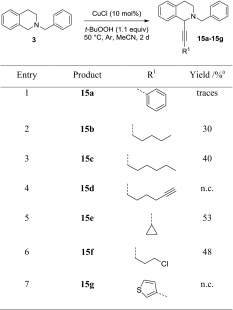
Substrate scope investigations of alkyne coupling partners

Standard conditions: 0.4 mmol **1**, 0.2 mmol alkyne, 0.22 mmol *t*-BuOOH (~5.5 M solution in decane), 10 mol% CuCl, 1 cm^3^ MeCN, and argon atmosphere

^a^Isolated yield

Since our overall goal was to develop a more convenient protocol, we wanted to test whether the so far applied oxidant could be substituted for a cheaper, more readily available one. Before screening for other oxidants, we tested the role of the oxidant by applying various amounts of Cu(I) and Cu(II) with or without external oxidant. As test reaction, again the alkynylation of 1 with phenylacetylene was used (Table 5).

**Table 5:** Role of oxidant

Entry	Catalyst	Loading	*t*-BuOOH	Yield/%^a^
1	CuCl	5 mol%	Yes	90
2	CuCl	5 mol%	No	–
3	CuCl_2_	5 mol%	Yes	78
4	CuCl_2_	5 mol%	No	Traces
5	CuCl	1 equiv.	No	–
6	CuCl_2_	1 equiv.	No	35

^a^GC yield with dodecane as internal standard

Traces of alkynylation product were detected via GC–MS in the presence of 5 mol% of copper(II) source (Table 5, entry 4) without *t*-BuOOH. When carrying out the reaction with 1 equivalent of copper(II) source (Table 5, entry 6), 35% of product **2** was detected after 2 d. No product was found to be formed with 5 mol% of copper(I) source and without *t*-BuOOH (Table 5, entry 2). In the presence of *t*-BuOOH, the reactions worked both with catalytic amounts (5 mol%) of copper(I) or copper(II) sources (Table 5, entries 1 and 3). These findings indicate that Cu(II) is the species which is needed for the alkynylation process and that it is reduced during the alkynylation reaction. Since the reaction works without *t*-BuOOH but with quantitative amount of Cu(II), the only role of *t*-BuOOH is to oxidize the copper source back to oxidation state II after the alkynylation step. Next, different oxidants were tested in our protocol (Table 6).

**Table 6:** Oxidant screening

Entry	Oxidant	P/bar	Yield **2**/%^a^	**16**
1	*t*-BuOOH	1	90	Traces
2	H_2_O_2_	1	–	–
3	O_2_	1	95	0.3 equiv.
4	Air	1	20	Traces
5	Air	5	93	Traces

^a^GC yield with dodecane as internal standard

Use of hydrogen peroxide did not lead to product formation, but decomposition of the substrate to several unidentifiable side products was observed (Table 6, entry 2). Performing the reactions under oxygen atmosphere led to almost quantitative formation of the desired alkynylation product **2** and formation of approximately 30% of oxidized product **16**, due to the excess of starting material (Table 6, entry 3). When carrying out the reactions under air atmosphere, only 20% of relative product formation was observed (Table 6, entry 4), either due to bad circulation in a closed vial with an air balloon or due to evaporation of the solvent and alkyne source when carrying out the reaction in an open vial. Performing the reactions under pressure (5 bar), almost quantitative conversion to **2** was observed (Table 6, entry 5), with only negligible amounts of **16** being observed. This is one rare example where air can be used as sole oxidant in a CDC reaction [23–25].

Since the reaction worked well in acetonitrile, we wanted to take it one step further toward an environmentally benign protocol by substituting acetonitrile by a green solvent, ideally water. To our delight, the reaction worked with identical efficiency also in water, even at shorter reaction time of 24 h and lower catalyst loading of 5 mol%! Additionally, a 1:1 ratio between **1** and alkyne source could be used instead of the formerly applied ratio of 2:1. Table 7 summarizes the results.

**Table 7:**
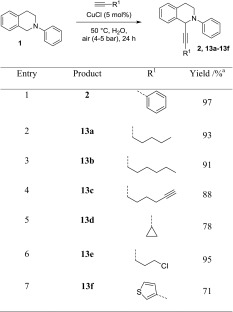
CDC alkynylation in water using air as oxidant

Standard conditions: 0.4 mmol **1**, 0.4 mmol alkyne, air (4–5 bar), 5 mol% CuCl, and 1 cm^3^ H_2_O

^a^Isolated yield

It can be seen that for all applied alkynes, excellent yields were obtained using this environmentally absolutely benign protocol. For phenylacetylene (Table 7, entry 1), 1-hexyne (entry 2), 1-octyne (entry 3), and 5-chloropent-1-yne (entry 6) yields greater than 90% were isolated. The drawback is the limited compatibility with other N-protecting groups. Interestingly, neither with the PMP-protected substrate **12** nor the benzyl-protected starting material **3,** significant conversions were detected in any example.

All our protocols disclosed here have one additional limitation. The alkyne scope is limited to non-volatile liquid or solid alkyne sources. To introduce also short-chained aliphatic alkynes, which are typically gaseous at room temperature, we recently disclosed a decarboxylative protocol, also taking advantage of copper catalysis [26]. Since the decarboxylative reaction (Scheme 2) worked well with the *t*-BuOOH/Ar/MeCN protocol, further experiments were carried out with the water/air protocol (see Scheme 2).

**Figure:**
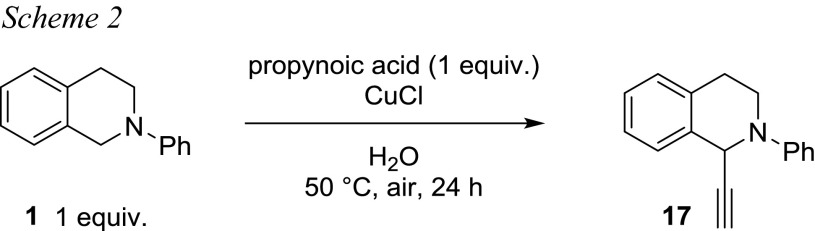

Yet, only traces of product **17** could be detected via GC–MS. Assuming that the decarboxylation process does not tolerate water as a solvent, reactions were carried out in MeCN, leading to higher conversion, but with no reproducible yields due to the instability of the pressure vial sealing in the presence of MeCN and high pressure. Further reactions were carried out in different water/MeCN mixtures, as listed below in Table 8.

**Table 8:** Solvent screening for decarboxylative coupling in aqueous media

Entry	Ratio H_2_O/MeCN	Ratio TIQ/alkyne	Yield **17**/%
1	100:0	2:1	Traces
2	99:1	2:1	Traces
3	90:10	2:1	Traces
4	50:50	2:1	Traces
5	100:0	1:1	Traces
6	99:1	1:1	5^a^
7	90:10	1:1	50^b^
8	50:50	1:1	80^b^

^a^GC yield with dodecane as internal standard

^b^Isolated yield

When the typical ratio of TIQ substrate:alkyne source of 2:1 was used, only traces of product **17** were formed independent of the solvent composition (Table 8, entries 1–4). However, when changing to a 1:1 ratio, the situation changed and at a water/MeCN ratio of 50:50, an isolated yield of 80% could be achieved (Table 8, entry 8). Ratios with higher MeCN content resulted in decomposition of the sealing.

Using **1** as substrate, excellent results were obtained for the two longest chained alkynoic acids (Table 9, entries 4 and 5), which gave almost quantitative yield. Also propynoic acid gave a good yield of **17** of 80% (Table 9, entry 1). Butynoic acid and pentynoic acid gave only a mediocre yield of **18** and **19**, respectively (Table 9, entries 2 and 3).

**Table 9:** Decarboxylative coupling in aqueous media

Entry	Product	R^1^	Yield/%^a^
1	**17**	H	80
2	**18**	CH_3_	32
3	**19**	C_2_H_5_	47
4	**20**	*n*-C_3_H_7_	95
5	**13a**	*n*-C_5_H_11_	98

Standard conditions: **1** (0.4 mmol), alkynoic acid (0.4 mmol), CuCl (0.04 mmol), water:MeCN (1:1, 1 cm^3^), air, 50 °C, 24 h

^a^Isolated yield

## Conclusion

Summarizing, three different alkynylation methods were established on *N*-phenyl, *N*-PMP, and *N*-benzyl-1,2,3,4-tetrahydroisoquinoline. First, the parameters of Li’s protocol [21] were changed to a different solvent (MeCN) and catalyst (CuCl). Under these conditions, it was possible to introduce different alkynes to *N*-phenyl-, *N*-PMP-, and also *N*-benzyl-TIQ substrates **1**, **3**, and **12** in moderate to high yields.

Second, in the search for a greener process, it was possible to change the reaction conditions to water as solvent and air as oxidant (instead of MeCN and *t*-BuOOH). The water protocol was shown to be only applicable to *N*-phenyl-TIQ **2**, leading to higher yields under greener conditions than with the original procedure in MeCN.

Third, to introduce also shorter alkynes and terminal alkynes to the C1-position, a decarboxylative protocol was developed, using alkynoic acids as alkyne sources, air as oxidant, and water/MeCN as the solvent mixture.

## Experimental

Unless otherwise noted, chemicals were purchased from commercial suppliers and used without further purification. Flash column chromatography was performed on silica gel 60 from Merck (40–63 μm), whereas separations were carried out using a Büchi SepacoreTM MPLC system. For TLC, aluminum-coated silica gel was used and signals were visualized with UV light (254 nm). GC–MS runs were performed on a Thermo Finnigan Focus GC/DSQ II using a standard capillary column BGB 5 (30 m × 0.32 mm ID) and the following settings were used as standard: injection: 1 mm^3^ (hot needle-technique), split-injection (split-ratio: 1:8); flow: 2 cm^3^/min helium; injector block temperature: 250 °C; MS-transferline temperature: 280 °C. HR-MS was carried out by E. Rosenberg at the Vienna University of Technology, Institute for Chemical Technologies and Analytics. All samples were analyzed by LC–IT-TOF-MS in only positive ion detection mode upon recording of MS and MS/MS spectra. For the evaluation in the following, only positive ionization spectra were used (where the quasi-molecular ion is the one of [M+H]^+^), and further data or information were not taken into consideration. Melting points were determined using a Kofler-type Leica Galen III micro hot stage microscope. ^1^H NMR and ^13^C NMR spectra were recorded on a Bruker AC 200 (200 MHz) or on a Bruker Avance UltraShield 400 (400 MHz) spectrometer. Chemical shifts are reported as ppm downfield from TMS (tetramethylsilane) as internal standard with multiplicity, number of protons, allocation, and coupling constant(s) in Hertz.

### General Procedure A

A mixture of 2 mg copper(I) chloride (0.02 mmol, 0.1 equiv.) and the corresponding 1,2,3,4-tetrahydroisoquinoline (0.4 mmol, 2.0 equiv.) in 1 cm^3^ MeCN was flushed with Ar for about 2 min and then 0.04 cm^3^ *tert*-butyl hydroperoxide (5.5 M in decane) was dropped into the mixture via syringe at room temperature, followed by the alkyne (0.2 mmol, 1.0 equiv.). The reaction temperature was raised to 50 °C and the mixture was stirred at this temperature for 2 days and then cooled to room temperature. The resulting suspension was diluted with diethyl ether or dichloromethane and filtered through a little amount of silica gel in a frit. The solvent was evaporated and the residue was purified by column chromatography or preparative TLC.

### General Procedure B

To a mixture of 4 mg copper(I) chloride (0.04 mmol, 0.1 equiv.) and the corresponding 1,2,3,4-tetrahydroisoquinoline (0.4 mmol, 1.0 equiv.) in 1 cm^3^ water in a pressure vial, the alkyne (0.4 mmol, 1.0 equiv.) was added. The vial was quickly filled with air to a pressure of 4–5 bar. The reaction mixture was then stirred at 50 °C for 24 h. After cooling down to room temperature, the reaction mixture was extracted 3× with 2 cm^3^ EtOAc, the organic phases were combined, the solvent was evaporated, and the residue was purified by column chromatography or preparative TLC.

### General Procedure C

To a mixture of 4 mg copper(I) chloride (0.04 mmol, 0.1 equiv.), 83.7 mg 2-phenyl-1,2,3,4-tetrahydroisoquinoline (0.4 mmol, 1.0 equiv.) in 1 cm^3^ of a 1:1 mixture of water and MeCN in a pressure vial, alkynoic acid (0.4 mmol, 1.0 equiv.) was added. The vial was quickly filled with air to a pressure of 4–5 bar. The reaction mixture was then stirred at 50 °C for 24 h. After cooling down to room temperature, the solvent was evaporated and the residue was purified by column chromatography or preparative TLC.

#### *N*-*Phenyl*-*1,2,3,4*-*tetrahydroisoquinoline* (**1**)

Copper(I) iodide (39.8 mg, 0.21 mmol, 0.1 equiv.) and 887.3 mg potassium phosphate (4.18 mmol, 2.09 equiv.) were weighed in a round flask which was evacuated and back filled with nitrogen three times. 2-Propanol (2 cm^3^), 0.23 cm^3^ ethylene glycol, 426.4 mg iodobenzene (0.23 cm^3^, 2.09 mmol, 1.05 equiv.) and 0.27 g 1,2,3,4-tetrahydroisoquinoline (0.26 cm^3^, 2.0 mmol, 1 equiv.) were added via micro syringe at room temperature. The reaction mixture was heated to 85–90 °C, stirred for 24 h and then allowed to cool to room temperature. Diethyl ether (5 cm^3^) and 5 cm^3^ water were then added to the reaction mixture. The organic layer was extracted by diethyl ether (2 × 20 cm^3^). The combined organic phases were washed with brine and dried over magnesium sulfate. The solvent was removed in vacuo and the crude mixture purified by column chromatography on silica gel (PE:EtOAc = 20:1) to give 83% (0.347 g, 1.66 mmol) of **1** as a beige solid. M.p.: 43–46 °C (lit. m.p.: 45–46 °C [27]); *R*_f_ = 0.69 (PE:EtOAc = 10:1).

#### *2*-*Phenyl*-*1*-*phenylethynyl*-*1,2,3,4*-*tetrahydroisoquinoline* (**2**)

It was prepared according to the General Procedure A (86%, 53 mg, 0.17 mmol) and B (97%, 120 mg, 0.38 mmol). The product was isolated by column chromatography (PE:DCM = 10:3) as a light yellow oil. NMR data were in agreement with the literature [21].

#### *N*-*Benzyl*-*1,2,3,4*-*tetrahydroisoquinoline* (**3**)

To an argon-degassed solution of 2.66 g THIQ (2.53 cm^3^, 20 mmol, 1.0 equiv.) and 6.07 g TEA (8.4 cm^3^, 60 mmol, 3.0 equiv.) in 50 cm^3^ dry DCM, 5.13 g benzyl bromide (3.4 cm^3^, 30 mmol, 1.5 equiv.) was added at 0 °C. After 10 min, the reaction mixture was warmed to r.t. and stirred under argon for 5 h. The reaction mixture was quenched with aqueous saturated sodium carbonate solution, and extracted three times with EtOAc. The collected organic layers were washed twice with brine, dried over sodium sulfate, filtered, and evaporated. The crude product was purified via column chromatography (PE:CHCl_3_ = 3:1) to give 82% (3.68 g, 16.5 mmol) of **3** as a pale yellow solid. M.p.: 35–37 °C (lit. m.p.: 35–36 °C [28]); TLC: *R*_f_ = 0.36 (PE:CHCl_3_ = 3:1).

#### *N*-*(4*-*Methoxybenzyl)*-*1,2,3,4*-*tetrahydroisoquinoline hydrochloride* (**4**)

To an argon-degassed solution of 1.33 g 1,2,3,4-tetrahydroisoquinoline (1.27 cm^3^, 10 mmol, 1.0 equiv.) and 3.04 g TEA (4.2 cm^3^, 30 mmol, 3.0 equiv.) in 15 cm^3^ dry DCM, 2.35 g 4-methoxybenzylchloride (2.03 cm^3^, 15 mmol, 1.5 equiv.) was added at 0 °C. After 10 min, the reaction mixture was warmed to r.t. and stirred under argon for 12 h. The reaction mixture was diluted with aqueous 2 M HCl and extracted three times with EtOAc. The collected organic layers were washed twice with brine, dried over sodium sulfate, filtered, and evaporated. The crude product was triturated in hot EtOAc, cooled down to −20 °C, and the colorless precipitate collected by filtration to give 86% (2.50 g, 8.63 mmol) of **4** after drying as colorless solid. M.p.: 210–212 °C (lit. m.p.: 211 °C [29]); TLC: *R*_f_ = 0.55 (PE:EtOAc = 3:1).

#### *N*-*Methyl*-*1,2,3,4*-*tetrahydroisoquinoline* (**5**)

1,2,3,4-THIQ (1.332 g, 10 mmol) was added, under cooling, to 2.302 g formic acid (50 mmol) and 0.751 g formaldehyde (25 mmol). The reaction mixture was refluxed overnight, diluted with 2 M hydrochloric acid, and then extracted with EtOAc. This solution was neutralized with brine and dried with sodium sulfate. The EtOAc was vaporized and the crude mixture separated via column chromatography (PE:EtOAc = 20:1) to give 87% (1.28 g, 8.7 mmol) of **5** as a yellow oil. *R*_f_ = 0.70 (PE: EtOAc = 10:1); NMR data were in agreement with the literature [30].

#### *N*-*(Pyridin*-*2*-*yl)*-*1,2,3,4*-*tetrahydroisoquinoline* (**6**)

1,2,3,4-Tetrahydroisoquinoline (666 mg, 0.63 cm^3^, 5.00 mmol, 1.0 equiv.) and 510 mg 2-fluoropyridine (0.45 cm^3^, 5.05 mmol, 1.05 equiv.) were placed in a screw-capped glass vial at r.t., heated to 120 °C, and stirred for 15 h. Completion of the reaction was monitored by TLC, the reaction mixture cooled to r.t., and directly subjected to flash column chromatography using gradient elution with PE:EtOAc (100:0–40:60) to afford the desired product **6** in 64% (670 mg, 3.19 mmol) as a pale yellow solid. M.p.: 39–42 °C; *R*_f_ = 0.65 (PE:EtOAC = 10:1); NMR data were in agreement with the literature [31].

#### *N*-*Boc*-*1,2,3,4*-*tetrahydroisoquinoline* (**7**)

To an argon-degassed solution of 2.66 g 1,2,3,4-tetrahydroisoquinoline (2.53 cm^3^, 20.0 mmol, 1.0 equiv.) and 6.07 g TEA (8.37 cm^3^, 60.0 mmol, 3.0 equiv.) in 45 cm^3^ dry DCM, a solution of 4.80 g Boc_2_O (5.05 cm^3^, 22.0 mmol, 1.1 equiv.) in 5 cm^3^ DCM was added dropwise. The reaction was stirred under argon atmosphere at r.t. for 15 h. Then, the solvent was evaporated in vacuo, and the residue directly subjected to flash column chromatography using gradient elution with PE:Et_2_O (100:0–40:60) to afford the desired product **7** in 99% (4.60 g, 19.7 mmol) as a colorless solid. M.p.: 27–35 °C; TLC: *R*_f_ = 0.79 (PE:Et_2_O = 5:1); NMR data were in agreement with the literature [32].

#### *N*-*Acetyl*-*1,2,3,4*-*tetrahydroisoquinoline* (**8**)

A 50 cm^3^ flask was loaded with 1.51 g 1,2,3,4-tetrahydroisoquinoline (1.44 cm^3^, 11.3 mmol, 1.0 equiv.) and 1.19 g acetic acid anhydride (1.10 cm^3^, 11.3 mmol, 1.0 equiv.). The mixture was heated to 100 °C for 3 h. After 1 h another equivalent of acetic acid anhydride was added to the reaction. The reaction mixture was cooled to r.t. and diluted with 200 cm^3^ DCM. The organic layer was washed twice with 2 M aqueous NaOH to get rid of excess acetic acid, washed twice with brine, dried over sodium sulfate, filtered, and evaporated. The crude product was subjected to flash column chromatography using gradient elution with PE:EtOAc (100:0–50:50) to afford the desired product **8** in 75% (1.49 g, 8.50 mmol) as pale yellow crystals. M.p.: 44–46 °C (lit. m.p.: 45–46 °C [33]); *R*_f_ = 0.29 (PE:EtOAc = 10:1).

#### *N*-*Pivaloyl*-*1,2,3,4*-*tetrahydroisoquinoline* (**9**)

To an argon-degassed solution of 2.66 g 1,2,3,4-tetrahydroisoquinoline (2.53 cm^3^, 20.0 mmol, 1.0 equiv.) and 6.07 g TEA (8.37 cm^3^, 60.0 mmol, 3.0 equiv.) in 50 cm^3^ dry DCM, 3.61 g pivaloyl chloride (3.68 cm^3^, 30.0 mmol, 1.5 equiv.) was added slowly at 0 °C. Then, the reaction mixture was warmed to r.t. and stirred at r.t. under argon for 2 h. The reaction mixture was cooled to 0 °C, diluted with aqueous 2 N HCl, and extracted three times with Et_2_O. The collected organic layers were washed twice with 2 N NaOH, and once with brine, dried over sodium sulfate, filtered, and evaporated. The crude product was subjected to flash column chromatography using gradient elution with PE:Et_2_O (100:0–40:60) to afford the desired product **9** in 86% (3.75 g, 17.3 mmol) as a pale yellow solid. M.p.: 63–65 °C (lit. m.p.: 67–69 °C [34]); *R*_f_ = 0.47 (PE:EtOAc = 5:1).

#### *N*-*Benzoyl*-*1,2,3,4*-*tetrahydroisoquinoline* (**10**)

Benzoyl chloride (4.22 g, 30.0 mmol, 1.5 equiv.), was added slowly to a solution of 2.66 g THIQ (2.53 cm^3^, 20.0 mmol, 1.0 equiv.) and 6.07 g TEA (8.37 cm^3^, 60.0 mmol, 3.0 equiv.) in 50 cm^3^ dry DCM at 0 °C. The reaction mixture was warmed to r.t. after completion of the addition and stirred at r.t. under argon for 15 h. Then, the reaction mixture was cooled to 0 °C, diluted with aqueous 2 N HCl, and extracted three times with Et_2_O. The collected organic layers were washed twice with 2 N NaOH, and once with brine, dried over sodium sulfate, filtered, and evaporated. The crude product was subjected to flash column chromatography using gradient elution with PE:Et_2_O (100:0–40:60) to afford the desired product **10** in 98% (4.67 g, 19.7 mmol) as a pale yellow solid. M.p.: 125–127 °C (lit. m.p.: 127–129 °C [34]); *R*_f_ = 0.24 (PE:EtOAc = 5:1).

#### *N*-*(4*-*Methoxyphenyl)*-*1,2,3,4*-*tetrahydroisoquinoline* (**12**)

Copper(I) iodide (39.8 mg, 0.21 mmol, 0.1 equiv.), 887.3 mg potassium phosphate (4.18 mmol, 2.09 equiv.), and 489.1 mg 4-iodoanisole (2.09 mmol, 1.05 equiv.) were put into a round flask which was evacuated and back filled with nitrogen three times. 2-Propanol (2 cm^3^), 0.23 cm^3^ ethylene glycol, and 0.27 g 1,2,3,4-tetrahydroisoquinoline (0.26 cm^3^, 2.0 mmol, 1.0 equiv.) were added via Hamilton syringe at room temperature. The reaction mixture was heated to 85–90 °C, stirred for 24 h, and then allowed to cool to room temperature. Diethyl ether (5 cm^3^) and 5 cm^3^ water were then added to the reaction mixture. The organic layer was extracted by diethyl ether (2 × 20 cm^3^). The combined organic phases were washed with brine and dried over magnesium sulfate. The solvent was removed in vacuo and the product purified by column chromatography on silica gel (PE:EtOAc = 20:1) to give 79% (0.38 g, 1.58 mmol) of **12** as a colorless solid. M.p.: 89–91 °C; TLC: *R*_f_ = 0.56 (PE:EtOAc = 5:1); NMR data were in agreement with the literature [35].

#### *1*-*(Hept*-*1*-*yn*-*1*-*yl)*-*2*-*phenyl*-*1,2,3,4*-*tetrahydroisoquinoline* (**13a**, C_22_H_25_N)

It was prepared according to the General Procedure A (67%, 41 mg, 0.13 mmol), B (93%, 116 mg, 0.36 mmol), and C (98%, 118 mg, 0.40 mmol). The product was isolated by column chromatography (PE/DCM) as a light yellow oil. *R*_f_ = 0.57 (PE:EtOAc = 20:1); ^1^H NMR (200 MHz, CDCl_3_): *δ* = 0.78–1.02 (m, 3H, H5″), 1.22–1.56 (m, 6H, H2″-H4″), 1.99 (t, 2H, H1″), 2.86–3.27 (m, 2H, H4), 3.51–3.87 (m, 2H, H3), 5.45 (s, 1H, H1), 6.90 (m, 1H, H4′), 7.02–7.48 (m, 8H, H5–H8, H2′, H3′) ppm; ^13^C NMR (50 MHz, APT, CDCl_3_): *δ* = 13.9 (q, C5″), 17.9 (t, C1″), 21.6 (t, C4″), 27.9 (t, C2″), 28.2 (t, C4), 30.2 (t, C3″), 42.0 (t, C3), 50.5 (d, C1), 79.9 (s, C alkyne), 84.5 (s, C alkyne), 116.1 (d, C2′), 118.9 (d, C4′), 125.9 (d, C7), 126.9 (d, C6), 127.4 (d, C5), 128.6 (d, C8), 128.9 (d, C3′), 133.8 (s, C8a), 135.9 (s, C4a), 149.2 (s, C1′) ppm; HR-MS: *m*/*z* calculated [M+H]^+^ 304.2060, found 304.2064.

#### *1*-*(Oct*-*1*-*yn*-*1*-*yl)*-*2*-*phenyl*-*1,2,3,4*-*tetrahydroisoquinoline* (**13b**, C_23_H_27_N)

It was prepared according to General Procedure A (49%, 31 mg, 0.1 mmol) and B (91%, 116 mg, 0.36 mmol). The product was isolated by column chromatography (PE/DCM) as a light yellow oil. *R*_f_ = 0.62 (PE:EtOAc = 20:1); NMR data were in agreement with the literature [21].

#### *1*-*(Octa*-*1,7*-*diyn*-*1*-*yl)*-*2*-*phenyl*-*1,2,3,4*-*tetrahydroisoquinoline* (**13c**, C_23_H_23_N)

It was prepared according to General Procedure A (93%, 58 mg, 0.19 mmol) and B (88%, 110 mg, 0.34 mmol). The product was isolated by column chromatography (PE/DCM) as a light yellow oil. *R*_f_ = 0.47 (PE:EtOAc = 20:1); ^1^H NMR (200 MHz, CDCl_3_): *δ* = 1.45–1.68 (m, 4H, H2″, H3″), 1.99 (s, 1H, H6″), 2.09-2.31 (m, 4H, H1″, H4″), 2.99–3.27 (m, 2H, H4), 3.55–3.83 (m, 2H, H3), 5.50 (s, 1H, H1), 6.88–7.46 (m, 9H, H5-H8, H2′, H3′) ppm; ^13^C NMR (50 MHz, CDCl_3_): *δ* = 18.2 (t, C4″), 18.6 (t, C1″), 27.6 (t, C3″), 27.8 (t, C2″), 29.1 (t, C4), 43.4 (t, C3), 52.2 (d, C1), 68.7 (d, C6″), 79.9 (s, C alkyne), 84.9 (s, C alkyne, C5″), 117.0 (d, C2′), 119.9 (d, C4′), 126.5 (d, C7), 127.3 (d, C6), 127.6 (d, C5), 129.2 (d, C8), 129.4 (d, C3′), 134.4 (s, C4a), 136.3 (s, C8a), 150.0 (s, C1′) ppm; HR-MS: *m*/*z* calculated [M+H]^+^ 314.1903, found 314.1900.

#### *1*-*(Cyclopropylethynyl)*-*2*-*phenyl*-*1,2,3,4*-*tetrahydroisoquinoline* (**13d**, C_20_H_19_N)

It was prepared according to General Procedure A (42%, 23 mg, 0.08 mmol) and B (78%, 86 mg, 0.32 mmol). The product was isolated by column chromatography (PE/DCM) as a light yellow oil. *R*_f_ = 0.55 (PE:EtOAc = 20:1); NMR data were in agreement with the literature [36].

#### *1*-*(5*-*Chloropent*-*1*-*yn*-*1*-*yl)*-*2*-*phenyl*-*1,2,3,4*-*tetrahydroisoquinoline* (**13e**, C_20_H_20_ClN)

It was prepared according to General Procedure A (77%, 46 mg, 0.15 mmol) and B (95%, 118 mg, 0.38 mmol). The product was isolated by column chromatography (PE/DCM) as a light yellow oil. *R*_f_ = 0.45 (PE:EtOAc = 20:1); ^1^H NMR (200 MHz, CDCl_3_): *δ* = 1.83 (qui, ^3^ *J* = 6.5 Hz, 2H, H2″), 2.31 (dt, ^3^ *J* = 6.7 Hz, ^4^ *J* = 2.1 Hz, 2H, H3″), 2.87–3.23 (m, 2H, H4), 3.38–3.79 (m, 4H, H3, H2″) 5.46 (s, 1H, H1), 6.9 (dt, ^3^ *J* = 7.2 Hz, ^4^ *J* = 1.0 Hz, 1H, H4′), 7.08 (dd, ^3^ *J* = 8.7 Hz, ^4^ *J* = 1.0 Hz, 2H, H2′), 7.16-7.40 (m, 6H, H5–H8, H3′) ppm; ^13^C NMR (50 MHz, CDCl_3_): *δ* = 16.4 (t, C3″), 29.1 (t, C4), 31.5 (t, C2″), 43.2 (t, C1″), 43.7 (t, C3), 52.2 (d, C1), 80.5 (s, CA1), 83.2 (s, CA2), 116.9 (d, C2′), 119.8 (d, C4′), 126.4 (d, C7), 127.3 (d, C6), 127.5 (d, C5), 129.1 (d, C8), 129.3 (d, C3′), 134.3 (s, C8a), 135.9 (s, C4a), 149.9 (s, C1′) ppm; HR-MS: *m*/*z* calculated [M+H]^+^ 310.1357, found 310.1347.

#### *2*-*Phenyl*-*1*-*(thiophen*-*3*-*ylethynyl)*-*1,2,3,4*-*tetrahydroisoquinoline* (**13f**, C_21_H_17_NS)

It was prepared according to General Procedure A (57%, 36 mg, 0.11 mmol) and B (71%, 90 mg, 0.28 mmol). The product was isolated by column chromatography (PE/DCM) as a light yellow oil. *R*_f_ = 0.49 (PE:EtOAc = 20:1); ^1^H NMR (200 MHz, CDCl_3_): *δ* = 2.92–3.28 (m, 2H, H4), 3.55–3.87 (m, 2H, H3), 5.66 (s, 1H, H1), 6.84–7.48 (m, 12H, H5-H8, H2′-H4′, H2″, H3″, H4″) ppm; ^13^C NMR (50 MHz, CDCl_3_): *δ* = 29.2 (t, C4), 43.7 (t, C3), 52.5 (d, C1), 80.11 (s, C alkyne), 88.42 (s, C alkyne), 116.9 (d, C2′), 119.9 (d, C4′), 122.3 (d, C2″), 125.3 (s, C1″), 126.6 (d, C7), 127.5 (d, C6), 127.7 (d, C5), 128.9 (d, C3″), 129.2 (d, C8), 129.4 (d, C3′), 130.3 (d, C4″), 134.7 (s, C4a), 135.6 (s, C8a), 149.8 (d, C4′) ppm; HR-MS: *m*/*z* calculated [M+H]^+^ 316.1154, found 316.1143.

#### *1*-*(2*-*Phenylethynyl)*-*2*-*(4*-*methoxyphenyl)*-*1,2,3,4*-*tetrahydroisoquinoline* (**14a**)

It was prepared according to General Procedure A (42%, 29 mg, 0.08 mmol). The product was isolated by column chromatography (PE/DCM) as a light yellow oil. *R*_f_ = 0.30 (PE:EtOAc = 20:1); NMR data were in agreement with the literature [21].

#### *1*-*(Hept*-*1*-*yn*-*1*-*yl)*-*2*-*(4*-*methoxyphenyl)*-*1,2,3,4*-*tetrahydroisoquinoline* (**14b**, C_23_H_27_NO)

It was prepared according to General Procedure A (75%, 50 mg, 0.15 mmol). The product was isolated by preparative TLC (CHCl_3_) as an orange oil. *R*_f_ = 0.31 (PE:EtOAc = 20:1); ^1^H NMR (200 MHz, CDCl_3_): *δ* = 0.83 (t, ^3^ *J* = 6.4 Hz, ^3^ *J* = 6.4 Hz, 3H, H5″), 1.11–1.45 (m, CH_2_, 6H, H2″–H4″), 2.07(dt, ^3^ *J* = 6.8 Hz, ^3^ *J* = 6.9 Hz, ^4^ *J* = 1.9 Hz, 2H, H1″), 2.87 (td, ^3^ *J* = 16.3 Hz, ^4^ *J* = 3.5 Hz, ^4^ *J* = 3.5 Hz, 1H, H4), 3.09 (ddd, ^3^ *J* = 16.5 Hz, ^4^ *J* = 9.7 Hz, ^4^ *J* = 6.8 Hz, 1H, H4), 3.45–3.58 (m, 2H, H3), 3.77 (s, 3H, OCH_3_), 5.28 (s, 1H, H1), 6.86 (d, ^3^ *J* = 9.1 Hz, 2H, H3′), 7.04 (d, ^3^ *J* = 9.1 Hz, 2H, H2′), 7.10–7.30 (m, 4H, H5–H8) ppm; ^13^C NMR (50 MHz, APT, CDCl_3_): *δ* = 14.0 (q, C5″), 18.7 (t, C1″), 22.2 (t, C2″), 28.4 (t, C4″), 29.0 (t, C4), 30.9 (t, C3″), 44.0 (t, C3), 53.8 (q, OCH_3_), 55.5 (d, C1), 78.9 (s, C alkyne), 86.0 (s, C alkyne), 114.3 (d, C2′), 120.0 (d, C3′), 126.0 (d, C7), 126.9 (d, C6), 127.4 (d, C5), 129.0 (d, C8), 133.8 (s, C4a), 136.3 (s, C8a), 144.3 (s, C1′), 154.0 (s, C4′) ppm; HR-MS: *m*/*z* calculated [M+H]^+^ 334.2165, found 334.2162.

#### *1*-*(Oct*-*1*-*yn*-*1*-*yl)*-*2*-*(4*-*methoxyphenyl)*-*1,2,3,4*-*tetrahydroisoquinoline* (**14c**)

It was prepared according to General Procedure A (61%, 42 mg, 0.12 mmol). The product was isolated by preparative TLC (CHCl_3_) as a light yellow oil. *R*_f_ = 0.35 (PE:EtOAc = 20:1); NMR data were in agreement with the literature [21].

#### *1*-*(Octa*-*1,7*-*diyn*-*1*-*yl)*-*2*-*(4*-*methoxyphenyl)*-*1,2,3,4*-*tetrahydroisoquinoline* (**14d**, C_24_H_25_NO)

Prepared according to General Procedure A (25%, 17 mg, 0.05 mmol). The product was isolated by preparative TLC (CHCl_3_) as a light yellow oil. *R*_f_ = 0.30 (PE:EtOAc = 20:1); ^1^H NMR (200 MHz, CDCl_3_): *δ* = 1.48–1.57 (m, 4H, Alkyl-CH_2_), 1.99–2.02 (m, 1H, alkyne-CH), 2.13–2.29 (m, 4H, alkyl-CH_2_), 2.88–3.30 (m, 2H, TIQ-CH_2_), 3.52–3.65 (m, 2H, TIQ-CH_2_), 3.88 (s, 3H, OCH_3_), 5.38 (s, 1H, TIQ-CH), 6.91–7.40 (m, 8H, Ar–CH) ppm; ^13^C NMR (50 MHz, CDCl_3_): *δ* = 18.2 (t, C4″), 18.6 (t, C1″), 27.6 (t, C3″), 27.9 (t, C2″), 29.3 (t, C4), 44.3 (t, C3), 54.1 (q, OCH_3_), 55.9 (d, C1), 56.3 (OCH_3_), 68.7 (C1), 79.7 (s, CA1), 84.6 (d, C6″), 85.5 (s, CA2), 94.8 (s, C5″), 114.6 (d, C2′), 118.4, 120.3 (d, C3′), 126.3 (d, C7), 127.2 (d, C6), 127.7 (d, C5), 129.3 (d, C8), 132.6, 134.1 (s, C4a), 136.4 (s, C8a), 144.6 (s, C1′), 154.3 (s, C4′) ppm; HR-MS: *m*/*z* calculated [M+H]^+^ 344.2009, found 344.2006.

#### *1*-*(Cyclopropylethynyl)*-*2*-*(4*-*methoxyphenyl)*-*1,2,3,4*-*tetrahydroisoquinoline* (**14e**, C_21_H_21_NO)

It was prepared according to General Procedure A (93%, 56 mg, 0.19 mmol). The product was isolated by preparative TLC (CHCl_3_) as a yellow oil. *R*_f_ = 0.31 (PE:EtOAc = 20:1); ^1^H NMR (200 MHz, CDCl_3_): *δ* = 0.49–0.76 (m, 4H, H2″), 1.09–1.26 (m, 1H, C1″), 2.84–3.23 (m, 2H, H4), 3.45–3.68 (m, 2H, H3), 3.83 (s, 3H, OCH_3_), 5.33 (s, 1H, H1), 6.91 (d, ^3^ *J* = 9.1 Hz, 2H, H3′), 7.08 (d, ^3^ *J* = 9.1 Hz, 2H, H2′), 7.14–7.35 (m, 4H, H5–H8) ppm; ^13^C NMR (50 MHz, CDCl_3_): *δ* = 0.0 (d, C1″), 8.6 (t, C2″), 29.2 (t, C4), 44.3 (t, C3), 54.0 (q, OCH3), 55.9 (d, C1), 74.3 (s, C alkyne), 89.3 (s, C alkyne), 114.5 (d, C2′), 120.3 (d, C3′), 126.3 (d, C7), 127.2 (d, C6), 127.7 (d, C5), 129.3 (d, C8), 134.1 (s, C4a), 136.4 (s, C8a), 144.5 (s, C1′), 154.3 (s, C4′) ppm; HR-MS: *m*/*z* calculated [M+H]^+^ 304.1696, found 304.1694.

#### *1*-*(5*-*Chloropent*-*1*-*yn*-*1*-*yl)*-*2*-*(4*-*methoxyphenyl)*-*1,2,3,4*-*tetrahydroisoquinoline* (**14f**, C_20_H_20_ClN)

It was prepared according to General Procedure A (52%, 32 mg, 0.10 mmol). The product was isolated by preparative TLC (CHCl_3_) as a light yellow oil. *R*_f_ = 0.28 (PE:EtOAc = 20:1); ^1^H NMR (200 MHz, CDCl_3_): *δ* = 1.72–1.88 (m, 2H, H2″), 2.21–2.35 (m, 2H, H3″), 2.81–3.23 (m, 2H, H4), 3.34–3.59 (m, 4H, H3, H1″), 3.80 (s, 3H, OCH_3_), 5.32 (s, 1H, H1), 6.89 (d, ^3^ *J* = 9.1 Hz, 2H, H3′), 7.05 (d, ^3^ *J* = 9.1 Hz, 2H, H2′), 7.12–7.34 (m, 4H, H5–H8) ppm; ^13^C NMR (50 MHz, CDCl_3_): *δ* = 16.5 (t, C3″), 29.3 (t, C4), 31.7 (t, C2″), 43.8 (t, C1″), 44.3 (t, C3), 54.3 (q, OCH_3_), 55.9 (d, C1), 77.8 (s, C alkyne), 80.4 (s, C alkyne), 114.7 (d, C2′), 120.4 (d, C3′), 126.4 (d, C7), 127.3 (d, C6), 127.7 (d, C5), 129.3 (d, C8), 134.1 (s, C4a), 136.0 (s, C8a), 154.6 (s, C4′) ppm; HR-MS: *m*/*z* calculated [M+H]^+^ 340.1463, found 340.1456.

#### *1*-*(Hept*-*1*-*yn*-*1*-*yl)*-*2*-*benzyl*-*1,2,3,4*-*tetrahydroisoquinoline* (**15b**, C_23_H_27_N)

It was prepared according to General Procedure A (30%, 19 mg, 0.06 mmol). The product was isolated by preparative TLC (CHCl_3_) as a light yellow oil. *R*_f_ = 0.65 (PE:CHCl_3_ = 3:2); ^1^H NMR (200 MHz, CDCl_3_): *δ* = 0.92 (t, 3H, H5″), 1.29–1.62 (m, 6H, H2″–H4″), 2.17–2.30 (m, 2H, H1″), 2.70–3.08 (m, 4H, H4, H3), 3.86 (dt, ^3^ *J* = 17 Hz, ^4^ *J* = 7.8 Hz, 2H, Ph-CH_2_), 4.56 (s, 1H, H1), 7.05–7.50 (m, 9H, H5–H8, H2′–H4′) ppm; ^13^C NMR (50 MHz, CDCl_3_): *δ* = 14.2 (q, C5″), 18.9 (t, C1″), 22.3 (t, C4″), 28.8 (t, C2″), 29.1 (t, C4), 31.2 (t, C3″), 45.7 (t, C3), 54.2 (t, Ph-CH_2_), 59.6 (d, C1), 78.0 (s, C alkyne), 87.3 (s, C alkyne), 125.8 (d, C7), 126.8 (d, C4′), 127.2 (d, C6), 127.8 (d, C5), 128.4 (d, C3′), 129.0 (d, C8), 129.4 (d, C2′), 133.9 (s, C4a), 136.4 (s, C8a), 138.6 (s, C1′) ppm; HR-MS: *m*/*z* calculated [M+H]^+^ 318.2216, found 318.2206.

#### *1*-*(Oct*-*1*-*yn*-*1*-*yl)*-*2*-*benzyl*-*1,2,3,4*-*tetrahydroisoquinoline* (**15c**)

It was prepared according to General Procedure A (40%, 27 mg, 0.08 mmol). The product was isolated by preparative TLC (CHCl_3_) as a yellow oil. *R*_f_ = 0.62 (PE:CHCl_3_ = 3:2); NMR data were in agreement with the literature [37].

#### *1*-*(Cyclopropylethynyl)*-*2*-*benzyl*-*1,2,3,4*-*tetrahydroisoquinoline* (**15e**, C_21_H_21_N)

It was prepared according to General Procedure A (53%, 30 mg, 0.11 mmol). The product was isolated by preparative TLC (CHCl_3_) as a light orange oil. *R*_f_ = 0.45 (PE:EtOAc = 20:1); ^1^H NMR (200 MHz, CDCl_3_): *δ* = 0.60–0.88 (m, 4H, H2″), 1.19–1.31 (m, 1H, H1″), 2.62–3.09 (m, 4H, H4, H3), 3.68–3.96 (m, 2H, Ph-CH_2_), 4.54 (s, 1H, H1), 7.01–7.56 (m, 9H, H5–H8, H2′–H4′) ppm; ^13^C NMR (50 MHz, CDCl_3_): *δ* = 0.0 (d, C1″), 8.8 (t, C2″), 29.3 (t, C4), 45.9 (t, C3), 54.4 (t, Ph-CH_2_), 59.7 (d, C1), 73.4 (s, C alkyne), 90.6 (s, C alkyne), 126.0 (d, C7), 127.0 (d, C4′), 127.40 (d, C6), 128.0 (d, C5), 128.6 (d, C3′), 129.2 (d, C8), 129.6 (d, C2′), 134.2 (s, C4a), 136.4 (s, C8a), 138.8 (s, C1′) ppm; HR-MS: *m*/*z* calculated [M+H]^+^ 288.1747, found 288.1738.

#### *1*-*(5*-*Chloropent*-*1*-*yn*-*1*-*yl)*-*2*-*benzyl*-*1,2,3,4*-*tetrahydroisoquinoline* (**15f**, C_21_H_22_ClN)

It was prepared according to General Procedure A (48%, 31 mg, 0.10 mmol). The product was isolated by preparative TLC (CHCl_3_) as a light yellow oil. *R*_f_ = 0.4 (PE:EtOAc = 20:1); ^1^H NMR (200 MHz, CDCl_3_): *δ* = 1.98 (qui, ^3^ *J* = 6.6 Hz, 2H, H2″), 2.46 (dt, ^3^ *J* = 6.8 Hz, ^4^ *J* = 2.0 Hz, 2H, H3″), 2.68–2.86 (m, 2H, H4), 2.88–3.01 (m, 2H, H3), 3.67 (t, ^3^ *J* = 6.4 Hz, 2H, H1″), 3.80 (d, ^3^ *J* = 13.2 Hz, 1H, Ph-CH_2_), 3.90 (d, ^3^ *J* = 13.2 Hz, 1H, Ph-CH_2_), 4.57 (s, 1H, H1), 7.05–7.49 (m, 9H, H5–H8, H2′–H4′) ppm; ^13^C NMR (50 MHz, CDCl_3_): *δ* = 16.5 (t, C3″), 31.8 (t, C2″), 43.9 (t, C1″), 45.8 (t, C3), 54.2 (t, Ph-CH_2_), 59.7 (d, C1), 79.3 (s, C alkyne), 85.2 (s, C alkyne), 125.9 (d, C7), 127.0 (d, C4′), 127.3 (d, C6), 127.8 (d, C5), 128.5 (d, C3′), 129.2 (d, C8), 129.4 (d, C2′), 134.1 (s, C4a), 136.2 (s, C8a), 138.5 (s, C1′) ppm; HR-MS: *m*/*z* calculated [M+H]^+^ 324.1514, found 324.1504.

#### *1*-*Ethynyl*-*2*-*phenyl*-*1,2,3,4*-*tetrahydroisoquinoline* (**17**, C_17_H_15_N)

It was prepared according to General Procedure C (80%, 75 mg, 0.32 mmol). The product was purified via preparative TLC (PE:CHCl_3_ = 3:1) as a colorless oil. *R*_f_ = 0.70 (CHCl_3_); ^1^H NMR (200 MHz, CDCl_3_): *δ* = 2.33 (d, 1H, HA2), 3.00 (m, 2H, H4), 3.59 (m, 2H, H3), 5.48 (s, 1H, H1), 6.91 (t, ^3^ *J* = 7.2 Hz, 1H, H4′), 7.03–7.42 (m, 8H, H5-H8, H2′, H3′) ppm; ^13^C NMR (50 MHz, CDCl_3_): *δ* = 29.13 (t, C4), 43.42 (t, C3), 51.85 (d, C1), 73.07 (d, C alkyne), 83.18 (s, C alkyne), 116.87 (d, C2′), 120.13 (d, C4′), 126.66 (d, C7), 127.56 (d, C6), 127.73 (d, C5), 129.31 (d, C8), 129.51 (d, C3′), 134.59 (s, C4a), 135.16 (s, C8a), 149.61 (s, C1′) ppm; HR-MS: *m*/*z* calculated [M+H]^+^ 234.1277, found 234.1271.

#### *1*-*(Propyn*-*1*-*yl)*-*2*-*phenyl*-*1,2,3,4*-*tetrahydroisoquinoline* (**18**, C_18_H_17_N)

It was prepared according to General Procedure C (32%, 32 mg, 0.13 mmol). The product was purified via preparative TLC (PE:CHCl_3_ = 3:1) as a light yellow oil. *R*_f_ = 0.18 (PE:CHCl_3_ = 4:1); ^1^H NMR (200 MHz, DMSO-*d*_*6*_): *δ* = 1.77 (s, 3H, H1″), 2.90–3.22 (m, 2H, H4), 3.52–3.75 (m, 2H, H3), 5.42 (s, 1H, H1), 6.79–7.50 (m, 9H, H5–H8, H2′–H4′) ppm; ^13^C NMR (50 MHz, DMSO-*d*_*6*_): *δ* = 2.8 (q, C1″), 27.6 (t, C4), 41.6 (t, C3), 49.7 (d, C1), 78.4 (s, C alkyne), 79.7 (s, Calkyne), 115.3 (d, C2′), 118.2 (d, C4′), 125.5 (d, C7), 126.5 (d, C6), 126.9 (d, C5), 128.2 (d, C8), 128.5 (d, C3′), 133.4 (s, C4a), 135.4 (s, C8a), 148.5 (s, C1′) ppm; HR-MS: *m*/*z* calculated [M+H]^+^ 248.1434, found 248.1422.

#### *1*-*(Butyn*-*1*-*yl)*-*2*-*phenyl*-*1,2,3,4*-*tetrahydroisoquinoline* (**19**, C_19_H_19_N)

It was prepared according to General Procedure C (47%, 49 mg, 0.19 mmol). The product was purified via preparative TLC (PE:CHCl_3_ = 3:1) as an orange oil. *R*_f_ = 0.22 (PE:CHCl_3_ = 4:1); ^1^H NMR (200 MHz, CDCl_3_): *δ* = 1.10 (t, ^3^ *J* = 7.5 Hz, 3H, H2″), 2.91 (dq, ^3^ *J* = 7.4 Hz, ^4^ *J* = 1.8 Hz, 2H, H1″), 2.92–3.28 (m, 2H, H4), 3.60–3.86 (m, 2H, H3), 5.48 (s, 1H, H1), 6.94 (t, ^3^ *J* = 7.2 Hz, 1H, H4′), 7.09–7.44 (m, 8H) ppm; ^13^C NMR (50 MHz, CDCl_3_): *δ* = 12.81 (q, C2″), 14.29 (t, C1″), 29.13 (t, C4), 43.52 (t, C3), 51.94 (d, C1), 78.71 (s, C alkyne), 86.86 (s, C alkyne), 116.76 (d, C2′), 119.57 (d, C4′), 126.46 (d, C7), 127.29 (d, C6), 127.60 (d, C5), 129.15 (d, C8), 129.36 (d, C3′), 134.52 (s, C4a), 136.54 (s, C8a), 149.88 (s, C1′) ppm; HR-MS: *m*/*z* calculated [M+H]^+^ 262.1590, found 262.1584.

#### *1*-*(Pentyn*-*1*-*yl)*-*2*-*phenyl*-*1,2,3,4*-*tetrahydroisoquinoline* (**20**)

It was prepared according to General Procedure C (95%, 105 mg, 0.38 mmol). The product was purified via preparative TLC (PE:CHCl_3_ = 3:1) as an orange oil. *R*_f_ = 0.25 (PE:CHCl_3_ = 4:1); NMR data were in agreement with the literature [38].
